# Visual-language transformer-based tomato leaf disease detection for portable greenhouse monitoring device

**DOI:** 10.1186/s13007-025-01456-8

**Published:** 2025-10-28

**Authors:** Manveen Kaur, Rajmeet Singh, Shahpour Alirezaee, Irfan Hussain

**Affiliations:** 1 Department of Mechanical Engineering, University of Windsor, Windsor, Canada; 2 Department of Mechanical Engineering, Khalifa University, Abu Dhabi, UAE

**Keywords:** Tomato leave, Disease, BLIP-2, LoRA, VLM, LLM

## Abstract

Tomato leaf diseases pose a significant threat to global food security, necessitating accurate and efficient detection methods. This paper introduces the Tomato Leaf Disease Visual Language Model (TLDVLM), a novel approach based on the BLIP-2 architecture enhanced with Low-Rank Adaptation (LoRA), for precise classification of 10 distinct tomato leaf diseases. Our methodology integrates a sophisticated image preprocessing pipeline, utilizing GroundingDINO for robust leaf detection and SAM-2 for pixel-level segmentation, ensuring that the model focuses solely on relevant plant tissue. The TLDVLM leverages the powerful multimodal understanding of BLIP-2, with LoRA applied to its Q-Former module, enabling parameter-efficient fine-tuning without compromising performance. Comparative experiments demonstrate that the TLDVLM significantly outperforms baseline models, including CLIP-LoRA and ConvNeXT-tiny, achieving an accuracy of 97.27%, a precision of 0.9587, a recall of 0.9789, and an F1-score of 0.9681. Beyond classification, the finetuned TLDVLM checkpoints are integrated into a practical application for new image inference. This application displays the raw and segmented images, the predicted disease, and offers functionalities to fetch comprehensive information on disease causes and remedies using external APIs (e.g., OpenAI), with an option to download a PDF summary for offline access on a portable device. This research highlights the potential of LoRA-adapted Vision-Language Models in developing highly accurate, efficient, and user-friendly agricultural diagnostic tools.

## Introduction

Crop diseases pose a critical threat to global food security, particularly affecting staple crops such as wheat and tomatoes that form the backbone of agricultural economies worldwide. The economic significance of tomato production is substantial, with greenhouse cultivation in Canada alone contributing approximately 294,000 tonnes annually across 6,200 hectares, generating over 600 million CAD in revenue [1]. Similarly, the United Arab Emirates has made remarkable advances in tomato production by embracing controlled-environment agriculture (CEA) and sophisticated greenhouse technologies. These innovations address the unique challenges posed by the region’s arid climate, extreme temperatures, and scarce freshwater resources. Leading commercial operations like Pure Harvest Smart Farms exemplify this progress, achieving year-round production of up to 30 tomato varieties within 16-hectare climate-controlled facilities and generating yields exceeding 400 tonnes per hectare [2].

Globally, tomatoes *(Lycopersicon esculentum)* are cultivated across 4.582 million hectares and face persistent threats from diseases such as early and late blight, which cause substantial yield losses [3]. Traditional disease detection approaches, including manual field inspections and laboratory-based diagnostics, present significant limitations. These methods are labor-intensive, time-consuming, and often impractical for large-scale commercial farming operations [4–6].

Over the past decade, conventional machine vision techniques have emerged to address the limitations of manual detection methods while reducing labor costs. These systems rely on computational algorithms to segment diseased crop areas, extract relevant features, and classify plant diseases [7–9]. A notable example includes a K-means clustering algorithm enhanced with Particle Swarm Optimization (PSO) for segmenting early blight lesions in tomato images converted to hue-saturation-value (HSV) color space, achieving an F1 score of 0.90 [10]. However, conventional machine vision systems face inherent limitations due to their multi-stage algorithmic approach, which requires significant human expertise and design input, making their performance heavily dependent on human decision-making.

The rapid advancement of deep learning technology has led to widespread adoption of convolutional neural networks (CNNs) in agricultural disease detection [11, 12]. Researchers have successfully evaluated various CNN models for detecting diseases in apple leaves [13] and implemented deep learning solutions for diagnosing grape and sugarcane leaf diseases [14, 15].

One significant breakthrough involved developing a YOLOv3 (You Only Look Once) model capable of identifying 12 different diseases using 15,000 tomato images captured under natural field conditions, achieving a detection accuracy of 92.39%. Additionally, a U-Net-based segmentation approach demonstrated promising results when analyzing 1,408 tomato leaf images, achieving an F1 score of 0.91 [16]. Despite these advances, traditional deep learning models often struggle with real-world implementation challenges, including lighting variations, complex backgrounds, and the critical need to accurately localize specific regions of interest, such as diseased leaves, within complex images. To overcome these limitations, advanced object detection and segmentation models have been developed, including GroundingDINO and Segment Anything Model 2 (SAM-2). These models provide precise localization and detailed segmentation capabilities, significantly enhancing the ability to isolate relevant features for downstream analysis [17, 18]. GroundingDINO utilizes text prompts to achieve highly accurate object detection, while SAM-2 generates detailed segmentation masks that enable focused analysis of specific image regions [19]. These technological advances are particularly valuable for tomato disease detection applications, where accurately identifying and isolating affected leaves is crucial for reliable diagnosis.

Vision-Language Models (VLMs) represent a revolutionary approach that combines visual and textual processing capabilities, offering unprecedented potential for tasks requiring sophisticated understanding of both images and contextual information [20]. These models show particular promise for agricultural applications where interpretability and context matter significantly. Models such as BLIP-2 integrate visual and textual data to perform complex tasks including image captioning and visual question answering, making them exceptionally well-suited for interpreting complex agricultural imagery in conjunction with disease-specific prompts [21]. By fine-tuning BLIP-2 on carefully annotated datasets of tomato leaf images with corresponding disease labels, the model can learn to classify diseases based on visual patterns and contextual queries, such as "What disease is present in this tomato leaf?" [22]. This approach offers dual benefits: improved classification accuracy and interpretable outputs that align with human expertise, thereby facilitating broader adoption in practical agricultural settings. However, deploying VLMs in real-world scenarios presents several challenges, including the need for robust model fine-tuning, efficient management of large model checkpoints, and optimal utilization of computational resources [23]. Cloud-based platforms like Google Colab provide scalable infrastructure solutions for storing and processing large models such as BLIP-2, effectively addressing these deployment challenges.

This research introduces an innovative pipeline for tomato disease detection using a comprehensive visual language model (TLDVLM) that seamlessly integrates three key components:

- GroundingDINO for precise tomato leaf detection
- SAM-2 for detailed segmentation
- Fine-tuned BLIP-2 with LoRA for accurate disease classification

The entire workflow operates on Google Colab, where it retrieves the fine-tuned BLIP-2 model and processes input images through a systematic pipeline that segments and detects tomato leaves before performing disease classification using a text-guided methodology. The model generates comprehensive outputs including both concise and detailed disease descriptions, along with actionable treatment recommendations. The proposed TLDVLM system has been successfully implemented on portable devices specifically designed for greenhouse environment applications, demonstrating practical real-world deployment capabilities. This research contributes significantly to the field by presenting a scalable, automated solution for tomato disease detection that leverages the inherent strengths of VLMs to enhance both accuracy and interpretability, with broad potential applications in precision agriculture. The contributions of this work include:

- **Multi-Modal Vision-Language Architecture** We developed a novel end-to-end pipeline that integrates GroundingDINO object detection, SAM-2 instance segmentation, and a fine-tuned BLIP-2 visual-language model. This comprehensive framework successfully bridges computer vision and natural language processing technologies to create a robust solution for agricultural disease diagnostics.
- **Text-Guided Classification with Semantic Reasoning:** We implemented an advanced text-conditioned disease classification system that harnesses the semantic understanding capabilities of vision-language models to perform contextual disease identification. This approach represents a significant advancement beyond traditional feature-based classification methods, enabling interpretable, reasoning-driven predictions that align with human expertise.
- **Edge-Deployable VLM Framework:** We successfully designed and deployed a computationally efficient visual language model on portable hardware capable of real-time inference in greenhouse environments. This achievement demonstrates the practical feasibility of deploying large-scale VLMs in resource-constrained agricultural settings, bridging the gap between advanced AI research and practical agricultural applications.
- **Automated Disease Interpretation Pipeline:** We created a comprehensive end-to-end automated system that generates structured disease descriptions and actionable treatment recommendations without requiring human intervention. This system effectively addresses the semantic gap between visual disease detection and practical agricultural insights through sophisticated natural language generation capabilities.

## Related work

This section provides an extensive examination of existing literature on tomato disease classification. It further explores the implementation of vision-language models within agricultural applications and identifies current research limitations and opportunities for advancement.

### Existing methods for tomato disease classification

Historically, tomato disease diagnosis has relied heavily on manual assessment conducted by agricultural specialists. This traditional approach presents significant challenges, being both labor-intensive and susceptible to subjective interpretation errors that can compromise diagnostic accuracy. The reliance on human expertise creates bottlenecks in large-scale agricultural operations and introduces variability in disease identification across different specialists and conditions. Recent advances in machine learning and deep learning methodologies have revolutionized disease identification systems in tomato production, substantially improving both accuracy and operational efficiency. These technological developments have enabled the transition from subjective manual assessment to objective, data-driven diagnostic approaches that can operate at scale. Traditional machine learning methods for tomato disease diagnosis typically employ a two-stage process: manual feature extraction followed by classification using various algorithmic approaches. This methodology has demonstrated considerable success in controlled environments, though it requires significant domain expertise for effective feature engineering. Zhang et al. pioneered the use of color and texture features combined with support vector machines for tomato leaf disease classification, achieving reasonable accuracy on controlled datasets [24]. This approach established the foundation for feature-based disease identification by demonstrating that visual characteristics could be systematically quantified and used for automated classification. Building on this foundation, Brahimi et al. developed a comprehensive system utilizing traditional feature extraction methods, including local binary patterns and color histograms, followed by random forest classification for identifying various tomato diseases [25]. Their approach demonstrated the value of combining multiple feature types to improve diagnostic robustness. Kaur et al. advanced the field by leveraging traditional computer vision techniques combined with artificial neural networks to detect early and late blight in tomato leaves [26]. Their work showed improved accuracy compared to manual inspection methods, particularly in early-stage disease identification where timely intervention is most critical. The adoption of deep learning architectures has marked a significant advancement in automated plant disease diagnosis. ResNet models, in particular, have been extensively validated for tomato plant disease identification, demonstrating superior performance compared to traditional approaches [27, 28]. These deep architectures eliminate the need for manual feature engineering by automatically learning discriminative features directly from image data. The implementation of U-Net for segmentation-based leaf disease detection represents a significant methodological advancement [29]. This approach enables precise localization of diseased regions within leaf structures, providing more targeted diagnostic capabilities compared to whole-image classification methods. Recent innovations include the development of novel Attention Convolutional Bidirectional Gated Recurrent architectures combined with Modified Leaf in Wind Algorithms for comprehensive plant disease assessment and environmental monitoring in sustainable agriculture applications. These sophisticated models incorporate temporal dynamics and spatial attention mechanisms to improve diagnostic accuracy in real-world agricultural environments. The validation of computer vision algorithms extends beyond tomato disease detection, with comprehensive testing and validation performed on soybean leaf datasets for disease classification [30].

This cross-crop validation demonstrates the generalizability of deep learning approaches across different agricultural applications and establishes the broader potential of these technologies in precision agriculture. Contemporary research increasingly focuses on integrating disease detection systems with broader sustainable agriculture frameworks. These integrated approaches combine disease identification with environmental monitoring capabilities, enabling comprehensive crop health management systems that support both productivity and sustainability goals. Although these machine learning-based methodologies have demonstrated effectiveness, they frequently encounter limitations associated with the tedious and resource-intensive nature of manual feature extraction, leading to a transition toward deep learning-based solutions.

Recent comprehensive research has demonstrated the versatility of deep learning techniques across multiple health and agricultural domains. Systematic studies have explored deep learning contributions to brain health diagnosis, diabetes disease prediction, and plant disease identification, establishing a foundation for cross-domain knowledge transfer [31–34]. This interdisciplinary approach highlights the universal applicability of deep learning architectures in complex diagnostic tasks. Advanced neural network architectures have been specifically developed for agricultural monitoring applications. The Enhanced Convolutional Neural Network with Local Feature Correlation Pooling (ExCNN-LFCP) algorithm represents a significant advancement in plant growth monitoring within hydroponic systems [35]. This specialized approach demonstrates the adaptation of deep learning techniques to controlled agricultural environments. The development of the Gradient Weighted DenseNet-201 (GradWDN-201) model showcases sophisticated approaches to plant leaf disease classification across multiple crop types [36]. This model underwent comprehensive evaluation on four benchmark datasets including Corn, Banana, Rose, and Rice leaf diseases, with extensive preprocessing protocols to ensure optimal data quality and model performance.

Deep learning methodologies have garnered significant attention in tomato disease diagnosis by automating feature extraction processes, thereby delivering enhanced accuracy and improved system robustness compared to traditional manual approaches. This automation eliminates the subjectivity inherent in manual feature engineering while enabling the discovery of complex patterns that may not be apparent to human observers. Fuentes et al. pioneered the application of advanced object detection architectures by developing a deep learning model combining Faster R-CNN and Single Shot MultiBox Detector (SSD) to enhance tomato disease detection accuracy under challenging real-world acquisition conditions [37]. This work achieved significant improvements over traditional approaches by addressing practical deployment challenges including variable lighting conditions and complex field environments. Tm et al. conducted comprehensive evaluations of several CNN-based models, including AlexNet and VGG16, employing different training strategies to identify common tomato diseases such as early blight, late blight, and bacterial spot [38]. This systematic comparison provided valuable insights into the relative performance characteristics of different deep learning architectures for tomato disease classification tasks. Agarwal et al. developed sophisticated systems utilizing deep learning classifiers with particular emphasis on transfer learning approaches incorporating pre-trained models [39]. Their system accurately detects and classifies tomato diseases while achieving high accuracy and demonstrating significant potential for mobile deployment, addressing the practical need for field-deployable diagnostic tools. Abbas et al. advanced the field by developing a DenseNet-based methodology specifically optimized for tomato plant disease classification [40]. Their approach presented a computationally efficient and precise model capable of real-time disease detection that consistently surpassed existing approaches across various tomato disease datasets while demonstrating strong deployment feasibility in resource-constrained environments. Aversano et al. proposed an integrated framework that combines deep learning with ensemble learning techniques for enhanced tomato plant disease classification [41]. This approach achieved superior accuracy through advanced image preprocessing techniques and the collaborative utilization of multiple CNN architectures, demonstrating that ensemble methods can significantly improve diagnostic performance. Recent innovations include YOLOv5-based convolutional feature attention neural network algorithms specifically designed for plant disease classification [42, 43]. These approaches integrate attention mechanisms with state-of-the-art object detection frameworks, enabling more focused analysis of disease-relevant image regions while maintaining computational efficiency. These comprehensive investigations reveal both the significant advantages and inherent constraints of current deep learning models in agricultural applications. While these approaches have demonstrated remarkable accuracy improvements and automation capabilities, they also highlight the ongoing necessity for more resilient and adaptable solutions.

Contemporary research in tomato disease detection has witnessed a significant shift toward more sophisticated neural network architectures and innovative training strategies. This evolution reflects the field’s maturation from proof-of-concept demonstrations to practical, deployable solutions that address real-world agricultural challenges with enhanced accuracy and reliability. A groundbreaking advancement in practical agricultural technology came through the development of AI-powered mobile applications specifically designed for tomato disease detection [44]. This innovative approach utilized deep learning models optimized for edge deployment, directly addressing the critical practical deployment challenges encountered in diverse agricultural settings. The mobile application framework represents a paradigm shift from laboratory-based diagnostic systems to field-ready tools that agricultural practitioners can use directly in their operations. By optimizing deep learning models for mobile hardware constraints, this research bridged the gap between advanced AI capabilities and practical agricultural needs, enabling real-time disease detection without requiring specialized equipment or internet connectivity. Kawasaki et al. made significant contributions to greenhouse-based tomato disease detection by developing an enhanced EfficientNet model specifically optimized for controlled agricultural environments [45]. Their approach achieved remarkably high classification accuracies through systematic analysis of environmental factors that influence disease detection performance.

This research represents a sophisticated understanding of the interplay between environmental conditions and model performance in controlled agricultural settings. By analyzing factors such as lighting conditions, humidity levels, and plant growth stages within greenhouse environments, the researchers optimized their model to achieve superior performance specifically tailored to controlled cultivation conditions. The enhanced EfficientNet implementation demonstrates how modern deep learning architectures can be adapted and optimized for specific agricultural contexts, achieving improved accuracy while maintaining computational efficiency suitable for practical deployment in commercial greenhouse operations.

### Vision-language models in agriculture

Vision-Language Models (VLMs) represent a groundbreaking advancement in artificial intelligence, fundamentally transforming how machines understand and interact with the world. These sophisticated systems have revolutionized diverse applications by seamlessly integrating two critical cognitive capabilities: the ability to process and generate human-like text alongside comprehensive visual information comprehension. VLMs are built upon robust architectural foundations, with pioneering models such as CLIP (Contrastive Language-Image Pre-training) [46] and BLIP (Bootstrapping Language-Image Pre-training) [47] leading the technological advancement. These foundational architectures have established the framework for creating models that can effectively bridge the gap between visual perception and linguistic understanding.

In the field of agriculture, VLMs’ utilization has begun to be explored for various applications. PLLaMa, an open-source large language model based on LLaMA-2, specifically designed for plant science-related topics, demonstrating the potential of domain-specific language models in agricultural contexts was proposed in [48]. Kuska et al. [49] created AI-chatbots for agriculture to aid in interpreting decision support models in plant disease management, showcasing the integration of conversational AI with agricultural expertise. An application of large language models for answering agriculture-related questions, demonstrating significant promise in knowledge dissemination and decision support was proposed in [50]. Agriculture, with its unique environmental variables, disease manifestations, and regional nuances, requires datasets specifically tailored to local conditions and crop-specific characteristics for optimal accuracy. Existing works utilizing models like CLIP and BLIP in agricultural contexts highlight the need for datasets that accurately represent the diversity of real-world complexities inherent in tomato cultivation and disease manifestation.

A significant gap exists in incorporating VLMs into the operational workflows of agricultural practitioners, particularly those engaged in tomato cultivation. The agricultural workforce, characterized by varying technological expertise and widespread geographical distribution, requires intuitive, user-friendly interfaces and tools accessible under field conditions. Minimal research has addressed how VLMs can seamlessly integrate with agricultural workers to provide immediate, on-site diagnostic capabilities or management recommendations tailored specifically for tomato diseases.

Current VLM applications in agriculture often focus on general plant identification or broad agricultural question-answering, yet tomato disease diagnosis in field conditions demands a specialized approach that integrates domain-specific visual understanding with expert knowledge. The integration of vision foundation models like SAM (Segment Anything Model) with VLMs, as seen in precision farming applications, remains sparsely explored for tomato disease diagnosis. The potential of multimodal systems to combine detailed visual analysis of leaf symptoms with textual descriptions of disease progression and environmental factors for precise diagnoses represents an unexplored frontier. Furthermore, the application of parameter-efficient fine-tuning techniques such as Low-Rank Adaptation (LoRA) to VLMs for agricultural applications has received limited attention. These techniques offer the potential to adapt large-scale vision-language models to specific agricultural domains while maintaining computational efficiency, making them suitable for deployment in resource-constrained agricultural environments. The segmentation and localization of diseased areas within tomato leaves represents another underexplored area in VLM applications. While object detection approaches have been investigated, the integration of advanced segmentation models with VLMs for precise disease localization and classification has not been thoroughly explored in the context of tomato disease detection.

In summary, the literature reveals a distinct deficiency in utilizing VLMs for tomato disease diagnosis, further complicated by the requirement for agriculture-specific datasets, intuitive and accessible user interfaces, computationally efficient adaptation techniques, and comprehensive segmentation methodologies. The proposed research attempts to fill these voids, contributing to more accurate, accessible, and sustainable tomato cultivation practices through the application of advanced vision-language models combined with state-of-the-art segmentation techniques. Additionally, to the authors’ knowledge, no prior studies have documented the practical deployment of VLM-based tomato leaf disease detection systems in portable device for real-world agricultural settings.

## Methodology

This section outlines the experimental setup, data preprocessing, model architectures, fine-tuning strategies, and evaluation metrics employed in this study. It leverages BLIP-2 with Low-Rank Adaptation (LoRA) for efficient tomato leaf disease detection and classification, which is compared against baselines implemented with CLIP and ConvNeXT-tiny for further validation.

**Fig. 1:**
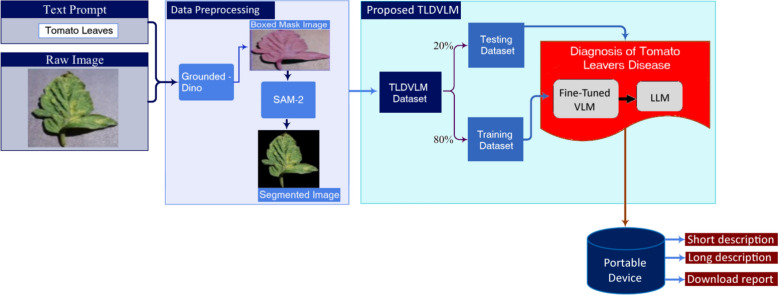
Overall workflow of tomato leave disease visual language model (TLDVLM)

Figure  1 illustrated the overall workflow of Tomato Leave Disease Visual Language Model (TLDVLM). The system architecture consists of four main components: (1) image and text prompt processing, (2) data preprocessing (3) proposed TLDVLM, and (4) deployment on portable device.

### Image and caption dataset

**Fig. 2:**
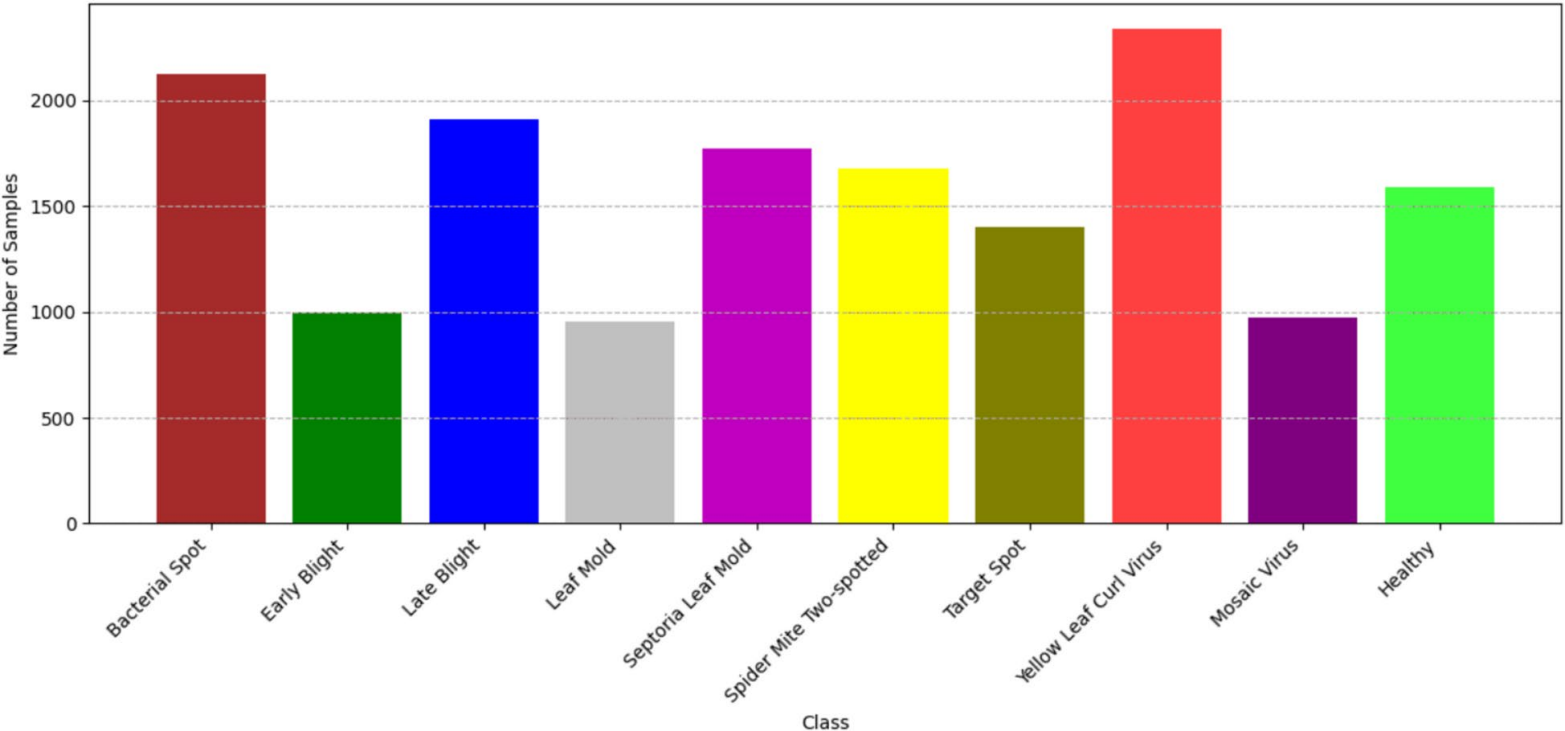
Dataset class distribution

The study utilized a comprehensive tomato leaf disease dataset sourced from Kaggle (Plant Village) and self capture dataset from Silal Greenhouse, Abu Dhabi. The dataset contains 10 distinct disease classes representative of common tomato pathologies. The dataset encompasses various disease conditions including Bacterial Spot, Early Blight, Late Blight, Leaf Mold, Septoria Leaf Spot, Spider Mites, Target Spot, Yellow Leaf Curl Virus, Mosaic Virus, And Healthy leaves. Each class contains varying numbers of images captured under different environmental conditions and lighting scenarios to ensure model robustness. Figure  2 illustrated the dataset class distribution. Table  1 mentioned the total amount of dataset images for training and validation of the model.

**Table 1:** Amount of dataset to train and validate the model

Dataset	PlantVillage	Own capture	Total
Dataset	13143	2000	15143

### Data preprocessing pipeline

Initial image processing involves resizing all input images to a standard 224$\times$224 pixels, followed by normalization of pixel values to the range [0,1] and standardization using ImageNet statistics. To enhance model generalization, data augmentation techniques such as random horizontal flips, rotations (±15 degrees), and color jittering are applied during training. To eliminate background noise and concentrate on the tomato leaf itself, we implement an automated leaf segmentation approach combining GroundingDINO and SAM-2.

GroundingDINO was chosen for its open-vocabulary, zero-shot detection capability, enabling flexible detection of tomato leaves specified by text prompts–crucial in agricultural environments where classes may vary or overlap. It demonstrates strong generalization, achieving 52.5 AP in COCO zero-shot tests [17]. In contrast, Detectron2 or Mask R-CNN are closed-set models requiring extensive retraining to recognize new classes. SAM-2 was selected for its prompt able, high-precision segmentation across complex scenarios. SAM-2’s performance has been recognized in systematic reviews of its enhanced segmentation efficacy and domain adaptability–covering fields like agriculture where variability is high [18]. Empirically, SAM-2 combined with YOLOv8 outperformed Mask R-CNN in apple segmentation tasks, boosting Precision, IoU, and F1 Score by 8.9%, 5.0%, and 5.6%, respectively. Even the original SAM (zero-shot, with post-processing) showed promising leaf-segmentation capability–achieving 60.3% precision and 63.2% recall without training–highlighting its value in data-scarce or annotation-limited scenarios, even if not perfect yet. Together, GroundingDINO’s flexible detection and SAM-2’s strong segmentation performance offers a powerful, adaptable pipeline–outperforming traditional fixed-class architectures in both generalization and efficiency in agricultural image analysis.

**Fig. 3:**
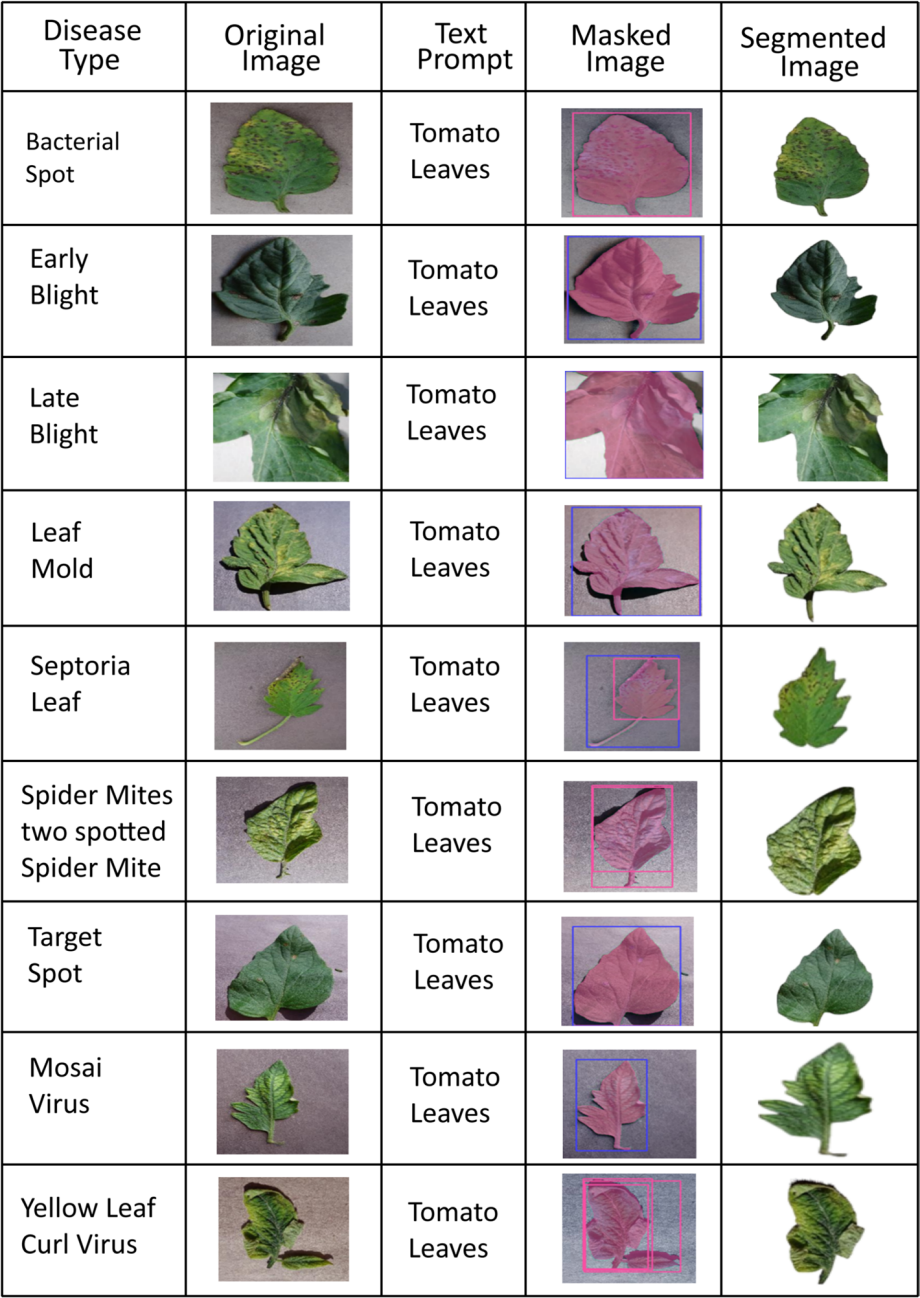
Segmentation results of GroundedDINO and SAM-2

GroundingDINO acts as the object detection component, identifying and localizing tomato leaves using text queries like "tomato leaf," providing flexible detection, precise bounding box generation, and multi-leaf handling. The Segment Anything Model 2 (SAM-2) then utilizes these bounding boxes as prompts to generate accurate pixel-level segmentation masks, thereby enabling background elimination and focusing classification solely on the plant tissue. Finally, these segmentation masks are applied to the original images, extracting leaf-only regions by masking out or replacing non-leaf pixels with a neutral background, ensuring the classification model focuses exclusively on disease-relevant features. Figure  3 displays the segmentation outcomes of GroundedDINO and SAM-2 across 10 distinct classes. GroundedDINO and SAM-2 leverage the contextual semantic information within images to accurately extract relevant tomato leaf characteristics while eliminating background interference.

The quality and consistency of the preprocessing steps involving GroundingDINO for detection and SAM-2 for segmentation were evaluated through both qualitative and quantitative analyses. Qualitatively, segmentation outputs were visually inspected across all 10 tomato leaf disease classes to confirm accurate leaf isolation and minimal background interference as shown in Fig. 3. This visual verification ensured that the masks consistently preserved disease-related features, such as lesion shape and color, which are critical for classification. Quantitatively, the downstream classification performance of the BLIP-2 LoRA model was compared with and without the GroundingDINO + SAM-2 preprocessing pipeline. The inclusion of preprocessing improved overall classification accuracy from 94.57% (ConvNeXt-tiny baseline without advanced segmentation) to 97.27% with preprocessing, alongside notable gains in precision, recall, and F1-score (Table 3). These improvements confirm that the preprocessing steps not only maintained but enhanced data quality for the classifier.

### Baseline models

This section will introduce the baseline models used for comparison against our proposed BLIP-2 LoRA (TLDVLM) method. For baseline model we used CLIP-LoRA and ConvNeXT-tiny models.

- **CLIP-LoRA**: CLIP (Contrastive Language-Image Pretraining) [51] is OpenAI’s vision-language model that learns to associate images and text through contrastive learning on large-scale datasets. For tomato leaf disease detection, the implementation leverages CLIP’s robust vision transformer component with Low-Rank Adaptation (LoRA) for efficient fine-tuning. It loads the pre-trained CLIP model (openai/clip-vit-base-patch 16), freezes its parameters to preserve learned features, and adds a custom classifier with layer normalization and dropout for 10 disease classes. The system uses LoRA with rank 16 to reduce trainable parameters, processes the tomato dataset with 224 $\times$ 224 image resizing and normalization, and trains for 20 epochs using Adam optimizer with mixed precision on GPU. The model achieves efficient adaptation to agricultural applications by fine-tuning only a small subset of parameters while maintaining CLIP’s powerful pre-trained representations. The CLIP-LoRA attained the accuracy around 91.87%.

- **ConvNeXT-Tiny**: ConvNeXt [52], developed by Meta AI, is a modern CNN architecture that incorporates transformer-inspired design principles like hierarchical feature maps, large kernel sizes, and layer normalization to compete with vision transformers while maintaining CNN efficiency. The implementation uses ConvNeXt-tiny as a backbone for tomato leaf disease classification, loading the pre-trained facebook/convnext-tiny-224 model with mixed precision and freezing all parameters except stages 2 and 3 for task-specific adaptation. The model trains for up to 20 epochs on A100 GPU using AdamW optimizer with differential learning rates (1e-6 for backbone, 3e-5 for classifier). This approach leverages ConvNeXt’s efficient architecture and pre-trained weights to achieve accurate tomato leaf disease classification with robust data augmentation and careful hyperparameter tuning. The ConvNext model achieved the accuracy around 94.57%.

### Proposed BLIP-2 LoRA method (TLDVLM)

In our study, we proposed the BLIP-2 LoRA method for classifying tomato leaf diseases using a custom dataset of 10 disease classes ( Plant-Village and own data capture from Silal Greenhouse, Abu Dhabi, UAE). The implementation incorporates Low-Rank Adaptation (LoRA) to fine-tune the Q-Former module, reducing computational overhead while preserving performance.

BLIP-2 was selected because its modular design- a frozen vision encoder, Q-Former for cross-modal representation learning, and frozen language model–allows highly parameter-efficient fine-tuning with LoRA while preserving strong multi-modal reasoning. In contrast, other VLMs like Flamingo is optimized for few-shot in-context learning with large interleaved image–text inputs, making it computationally heavier and less suited for constrained agricultural deployment scenarios [53] and MiniGPT-4 focuses on generative multi-modal interaction by aligning a frozen vision encoder with an advanced LLM, excelling in open-ended outputs like image description and creative tasks, but lacking the domain-specific discriminative efficiency required for disease classification [54].BLIP-2 thus offers the best balance of accuracy, efficiency, and adaptability for targeted, text-guided plant disease detection deployable on portable devices.

**Fig. 4:**
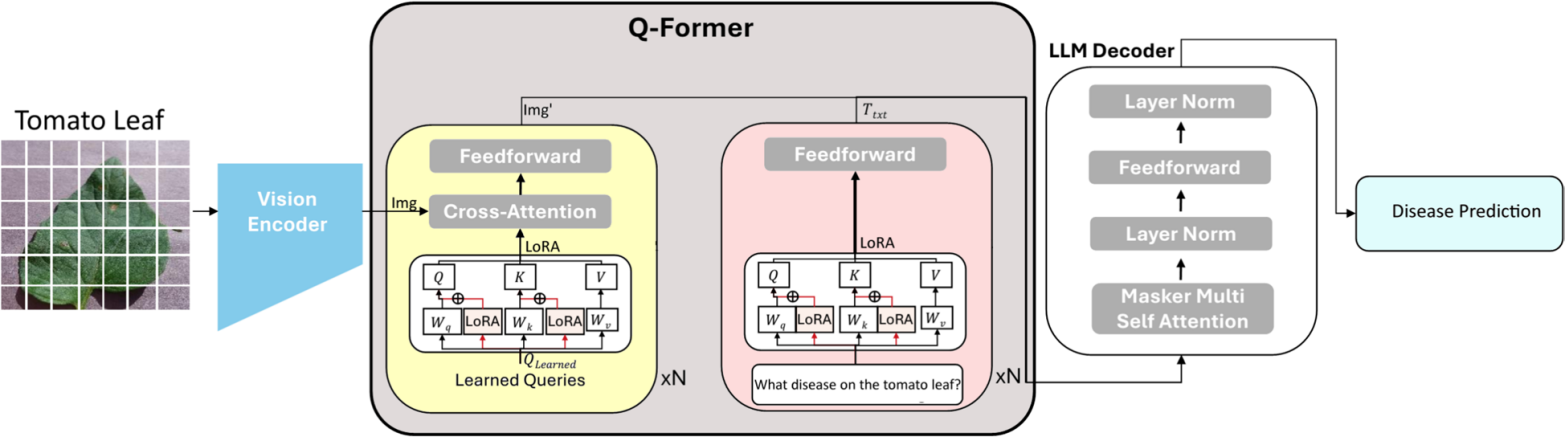
Architecture of BLIP-2 LoRA model

Figure  4 illustrated the architecture of the proposed BLIP-2 LoRA model. The architecture details an image classification system for specialized tasks, such as "Disease Prediction" on a "Tomato Leaf," by integrating Low-Rank Adaptation (LoRA) into a Vision-Language Model (VLM) framework, analogous to BLIP-2. The system processes an input image, segmenting it into patches that are then fed into a frozen Vision Encoder. This encoder, typically a pre-trained Vision Transformer, extracts high-level visual features (Img’) without undergoing further weight updates during fine-tuning. The core of this adapted VLM is the Q-Former, a multi-layered transformer module responsible for bridging the visual and linguistic modalities. The Q-Former contains distinct processing pathways for learned visual queries and textual prompts. Within the visual pathway, "Learned Queries" interact with the encoded image features (Img’) via cross-attention mechanisms, where the queries serve as the attention keys and values, and Img’ provides the query.

Crucially, LoRA is implemented within the linear projection layers of this cross-attention mechanism by introducing small, trainable low-rank matrices (LoRAQ,LoRAK,LoRAV) that are added to the frozen pre-trained weights. Similarly, the textual pathway, which processes an input prompt like "What disease on the tomato leaf?", also incorporates LoRA within its self-attention and feedforward networks, enabling efficient adaptation of the model’s linguistic understanding to the specific task. This selective fine-tuning of LoRA adapters significantly reduces the number of trainable parameters, thereby decreasing computational cost and memory footprint while preserving the vast pre-trained knowledge of the larger model. Finally, the refined multimodal features from the Q-Former are passed to a frozen LLM Decoder, which comprises multiple layers of masked multi-self-attention and feedforward networks. This LLM leverages its extensive language generation capabilities to produce the ultimate "Disease Prediction" based on the integrated visual and textual context provided by the LoRA-adapted Q-Former. This approach demonstrates a highly parameter-efficient strategy for specializing large VLMs for downstream image classification tasks, maintaining high performance while making fine-tuning more accessible.

In most VLM fine-tuning, LoRA (or PEFT) is applied to the language model and/or a small vision–language projector, while keeping the rest frozen [55, 56]. By contrast, our approach injects LoRA into BLIP-2’s Q-Former–the query transformer that learns a vision-to-language interface to a frozen LLM–so we adapt the cross-modal bridge itself rather than the LLM or only a projector. This leverages BLIP-2’s modular design (frozen vision encoder + frozen LLM + trainable Q-Former) to concentrate learning where alignment happens, yielding strong adaptation with very few trainable parameters (< 0.6M trainable params) and tailored for resource-constrained edge deployment, which is not reported in earlier agricultural LoRA applications.

Using LoRA with BLIP-2 for image classification offers substantial advantages by significantly reducing computational cost and memory footprint during fine-tuning, as it only trains a small set of low-rank matrices while freezing most pre-trained parameters [20, 55]. This efficiency not only makes fine-tuning accessible on more limited hardware but also accelerates the training process.

#### Training parameters

Model training was conducted on the Google Colab distributed computing platform using an NVIDIA A100 GPU with 40GB memory capacity. The optimization process employed the AdamW optimizer, with comprehensive hyperparameter configurations for the TLDVLM detailed in Table  2. The fine-tuning procedure demonstrated remarkable efficiency, completing within 5 h while consuming less than 40 GB of VRAM. This computational efficiency underscores the TLDVLM’s ability to effectively leverage limited datasets and optimize hardware utilization through the integration of Low-Rank Adaptation (LoRA) techniques. The final model size is 2.7B parameters for the base model plus 0.6 M parameters for the LoRA adapters and classifier.

**Table 2:** Hyperparameters for model tuning

Hyperparameter	Default Setting
Optimizer	AdamW
Batch Size	32
Epochs	20
Learning rate	1 e-7
Learning Schedule	Cosine Annealing

### Deployment on portable device

**Fig. 5:**
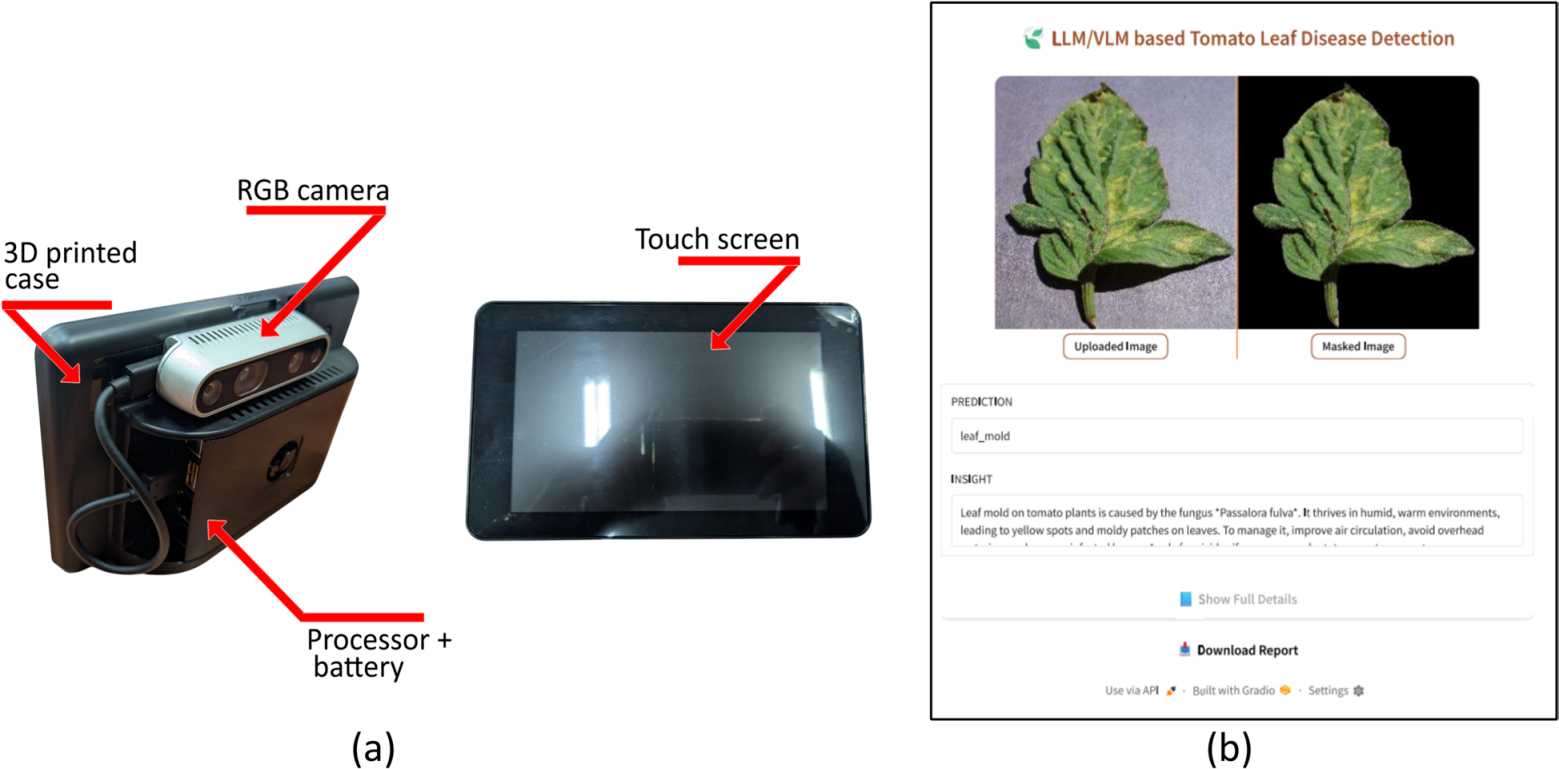
Proposed TLDVLM implementation: **a** Portable device, and **b** Gradio online user interface

Subsequently, the proposed model was deployed on a novel portable device designed for tomato leaf disease inspection within greenhouse environments. The model was transformed into a web-based application utilizing the Gradio online user interface platform. The portable device was manufactured at the Khalifa University Agrirobotics laboratory and validated in the Silal Greenhouse facility. Figure  5 illustrated the portable tomato leave disease detection device and webpage implementation of proposed TLDVLM on Gradio platform. The device includes the RGB camera, touch screen, Raspberry pi-5 processor, lithium battery, and 3D printed case.

**Fig. 6:**
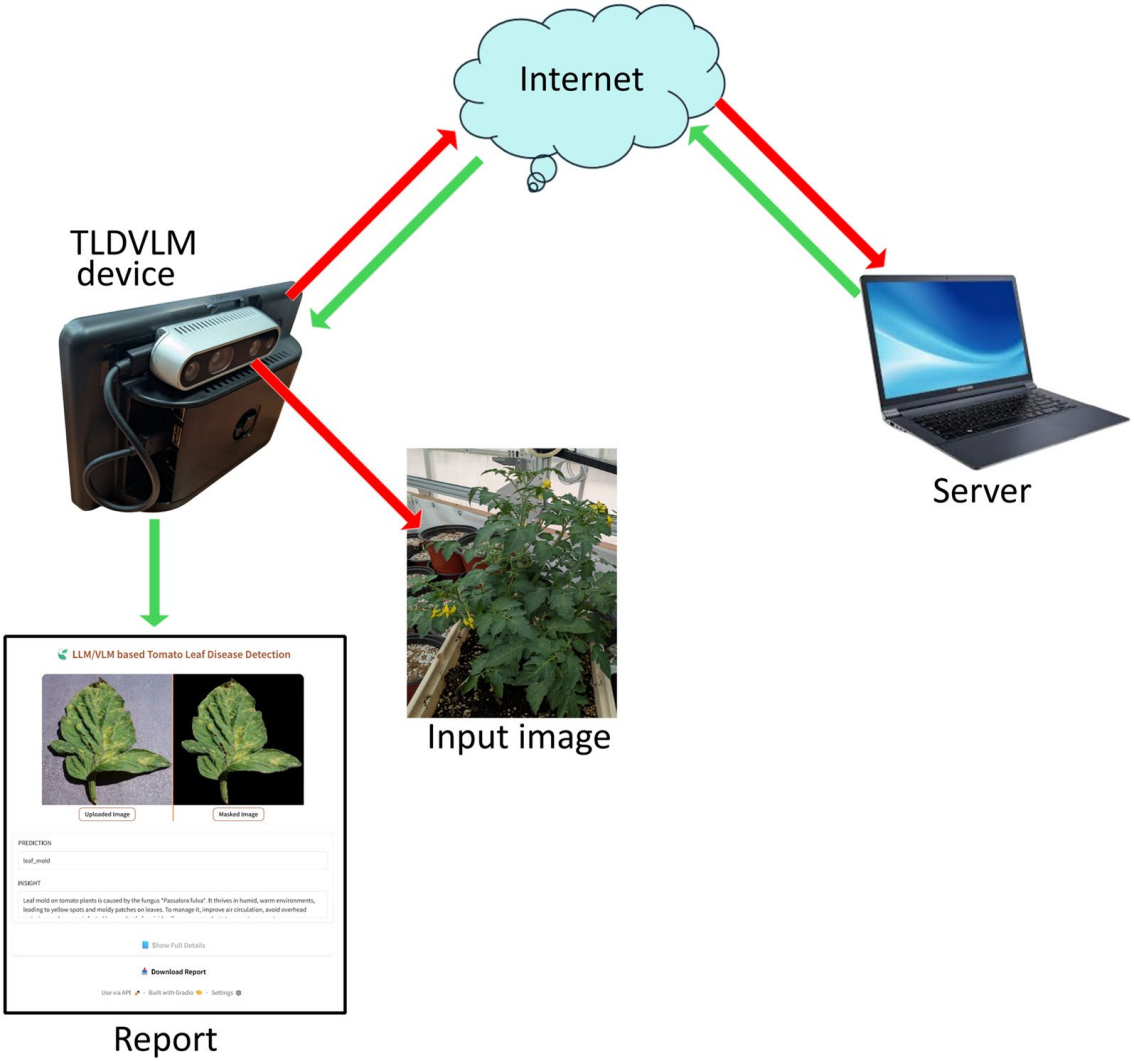
Proposed TLDVLM device communication system

The implemented system demonstrates comprehensive capabilities including segmentation, classification, and generation of both concise and detailed disease information along with corresponding treatment recommendations. Additionally, the system provides a comprehensive downloadable report in PDF format for documentation purposes. Figure  6 depicts the communication architecture of the TLDVLM device. The system captures leaf images and transmits them to a trained model server for classification analysis, subsequently receiving both concise and detailed descriptions of identified diseases along with corresponding remedial solutions. Additionally, a comprehensive report is generated to facilitate further analytical assessment. The device is connected on cloud server, so there is no issue of connection loss. Only internet servers are required to connect the device with main server. The plant scanning range is 20–30 cm.

## Results

This section presents the experimental results obtained from training and evaluating the proposed BLIP-2 with LoRA model and compares its performance against other baseline models: CLIP-LoRA and ConvNeXT-tiny. We also provide a detailed analysis of model convergence, class-specific performance, and overall robustness indicators. These results indicate that the model has successfully learned to distinguish between the 10 tomato disease classes with high accuracy while maintaining good generalization capabilities.

### Overall performance comparison

Table  3 shows the key performance metrics of proposed method with baseline models. As depicted from the Table  3 the BLIP-2 LoRA (proposed) model outperformed than others baseline models for detection of tomato leave diseases. The model demonstrated stable convergence, with a final training loss of 0.6068 and a validation loss of 0.6970 upon completion at 20 epoch. The small loss differential of 0.0902 suggests good generalization with very minimal overfitting. Performance metrics were robust: a high precision of 0.9587 indicates excellent accuracy in positive predictions, crucial for avoiding unnecessary treatments. An exceptional recall of 0.9789 highlights the model’s ability to identify nearly all actual disease instances, thereby minimizing missed diagnoses. The balanced F1-score of 0.9681 further confirms the model’s strong overall performance across all disease classes.

TLDVLM outperforms existing models by combining multimodal reasoning (BLIP-2) with precise segmentation (GroundingDINO + SAM-2), enabling higher accuracy (+2.7% over ConvNeXT-tiny, +5.4% over CLIP-LoRA) in complex greenhouse conditions. LoRA fine-tuning ensures <0.6M trainable parameters for efficient edge deployment and real time applications.

**Table 3:** Key performance metrics

Models	Accuracy	Precision	Recall	F1-Score
CLIP-LoRA	91.87%	0.9046	0.8896	0.8957
ConvNeXt-tiny	94.57%	0.9270	0.9463	0.9360
BLIP-2-LoRA (Proposed)	97.27%	0.9587	0.9789	0.9681

#### Confusion matrix analysis

**Fig. 7:**
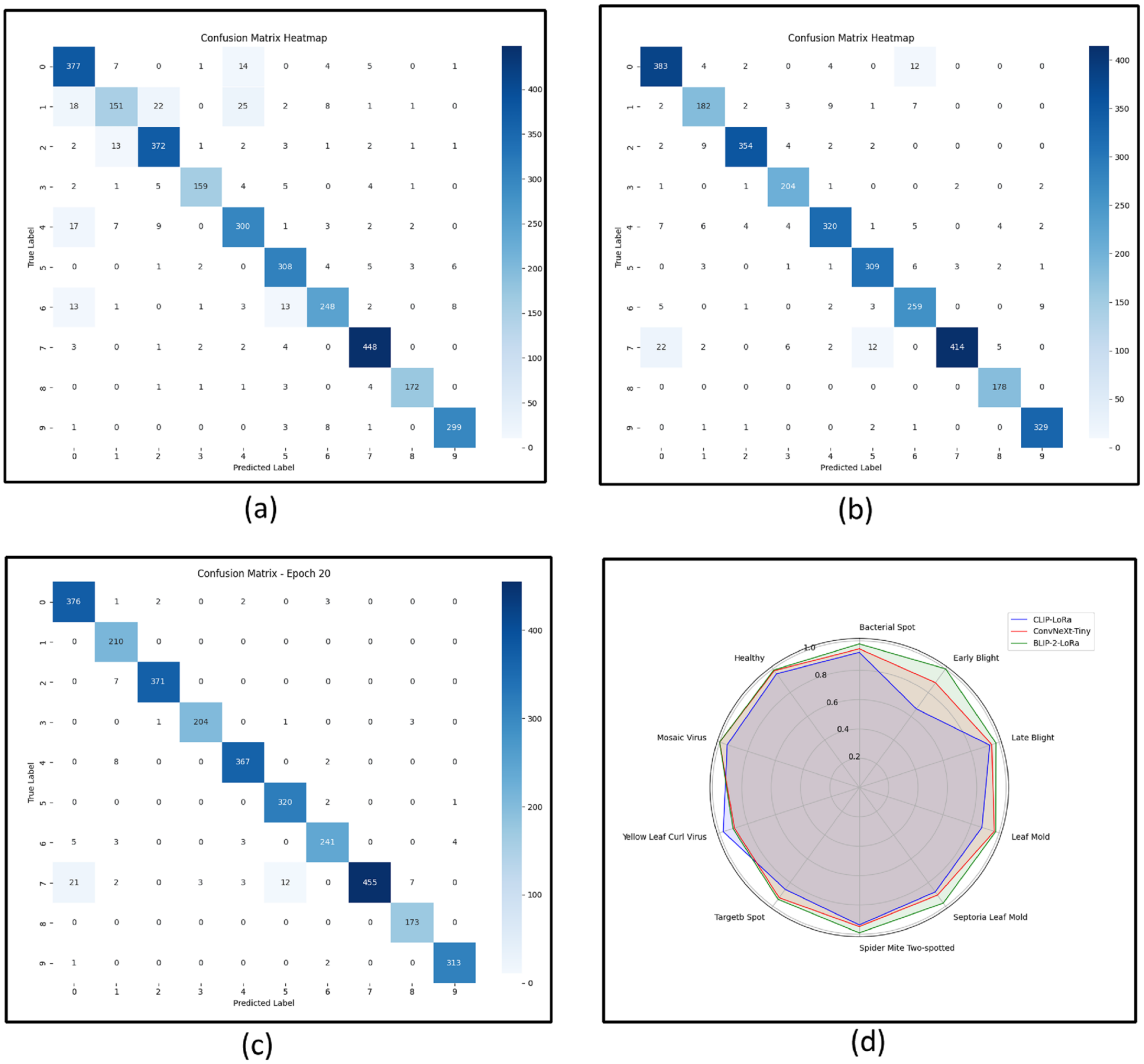
Confusion matrix: **a** CLIP-LoRA, **b** ConvNeXT-Tiny, **c** BLIP-2 LoRA, and **d** Radar plot

The confusion matrix reveals detailed insights into the model’s classification performance across all disease classes. Figure  7 illustrated the confusion matrix’s for CLIP-LORA, ConvNeXT-Tiny, and BLIP-2-LoRA. Figure  7a presented the heatmap for CLIP-LoRA baseline model across the 10 tomato leaf disease classes. The off-diagonal elements show a higher frequency of misclassifications compared to the BLIP-2 LoRA model (Figure  7c). For instance, class 1 (Early Blight) exhibits multiple misclassifications into other classes (e.g., 18 instances predicted as class 0, 22 as class 2, 25 as class 3), suggesting a less refined understanding of its visual features compared to TLDVLM. Class 6 also shows more dispersed errors. The overall lighter shades on the diagonal for several classes, along with more pronounced off-diagonal values, indicate that CLIP-LoRA’s discriminatory power is somewhat less precise than that of the BLIP-2 LoRA model for this specific dataset and task. Similarly, Fig.  7b represents the heatmap for ConvNeXT-Tiny baseline model. ConvNeXT-tiny generally performs well, the misclassifications patterns, indicated by off-diagonal values, are more prominent than those observed with BLIP-2 LoRA. For example, similar to CLIP-LoRA, there are noticeable misclassifications for several classes, such as class 1 (Early Blight) having 18 instances misclassified as class 0, and others scattering across different labels. Class 6 also demonstrates a notable number of incorrect predictions. The visual comparison with Fig.  7c highlights that while ConvNeXT-tiny, as a robust CNN, achieves good performance, it occasionally struggles with finer distinctions between disease types, leading to a higher incidence of misclassifications compared to the multi-modal TLDVLM.

Figure  7c illustrated the heatmap of BLIP-2 LoRA model. Notably, classes such as 1 (Early Blight), 4 (Leaf Mold), and 8 (Target Spot) show perfectly or near-perfectly classified instances with no or minimal off-diagonal values, confirming their highly distinctive visual features. Misclassifications, represented by non-zero off-diagonal entries, are generally low. The matrix reveals that the majority of errors occur between visually similar classes, with some instances of class 7 (Spider Mite Two-spotted) being misclassified across several other classes, suggesting subtle visual overlaps. The overall strong diagonal presence across all classes underscores the TLDVLM’s effective discrimination capabilities.

Figure  7d provides a comparative visualization of the F1-scores for each of the 10 tomato leaf disease classes across the three models: CLIP-LoRA (blue line), ConvNeXT-tiny (red line), and BLIP-2 LoRA (green line). Each spoke of the radar represents a specific disease class, with the F1-score ranging from 0 at the center to 1 at the outermost ring. The chart clearly illustrates that the BLIP-2 LoRA model (green line) consistently achieves the highest F1-scores across nearly all disease classes, encompassing a larger area within the radar plot. While ConvNeXT-tiny (red line) shows competitive performance on some classes, and CLIP-LoRA (blue line) performs reasonably, BLIP-2 LoRA’s green envelope consistently extends further towards the maximum F1-score of 1.0. This visual representation powerfully reinforces that the proposed TLDVLM with BLIP-2 LoRA demonstrates superior and more balanced classification performance across the entire spectrum of tomato leaf diseases compared to the other two baseline methods.

#### Evaluation metrics and performance assessment

**Fig. 8:**
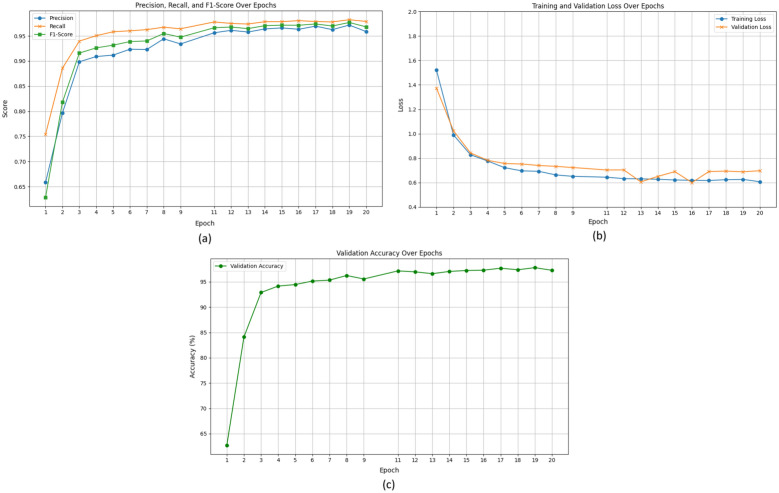
Evaluation metrics for BLIP-2 LoRA : **a** Precision, recall, F1 score, **b** Training and validation loss, and **c** Validation accuracy

Figure 8 illustrated the evaluation metrics of proposed (BLIP-2 LoRA) model for VLM based tomato leave disease classification and reasoning.

Figure  8a, displays the evolution of key classification metrics–Precision, Recall, and F1-Score–for the TLDVLM (BLIP-2 LoRA) model during training over 20 epochs. The x-axis represents the epoch number, and the y-axis denotes the score ranging from 0.6 to 1.0. All three metrics (Precision—blue circles, Recall—orange ’x’ markers, F1-Score—green squares) show a rapid increase during the initial epochs, signifying quick improvements in classification performance. After roughly epoch 6, the scores begin to stabilize and continue to gradually improve, reaching high plateaus. By epoch 20, the Precision, Recall, and F1-Score converge to approximately 0.9587, 0.9789, and 0.9681, respectively. The consistently high and closely aligned values across these metrics highlight the model’s balanced and robust performance in correctly identifying positive instances and minimizing both false positives and false negatives. Figure  8b, presents the progression of the training loss and validation loss for the TLDVLM (BLIP-2 LoRA) model over 20 epochs. Both the training loss (blue line with circular markers) and validation loss (orange line with ’x’ markers) demonstrate a sharp decrease during the initial epochs, indicating rapid learning. After approximately 4–5 epochs, the decrease in both curves stabilizes, suggesting convergence. The final training loss is observed to be around 0.6068, while the validation loss hovers around 0.6970. The consistently small gap between the training and validation loss curves throughout the training process indicates that the model is generalizing well to unseen data and is not significantly overfitting. This stable convergence confirms the effectiveness of the training configuration and regularization techniques employed. Figure  8c, tracks the Validation Accuracy for the TLDVLM (BLIP-2 LoRA) model throughout its 20 epochs of training. The x-axis denotes the epoch number, and the y-axis represents the accuracy percentage. The curve shows a steep increase in accuracy during the initial few epochs, rising from approximately 63% at Epoch 1 to over 90% by Epoch 3. The accuracy continues to climb steadily, eventually reaching a plateau above 95% around Epoch 8–10. From Epoch 10 onward, the validation accuracy remains consistently high, hovering between 96% and 97%, with minor fluctuations. This stable and high validation accuracy confirms the model’s robust learning and strong generalization capabilities on unseen data, indicating successful fine-tuning for the tomato leaf disease classification task.

Table  4 presents a comparative analysis of the proposed model against existing traditional approaches specifically designed for tomato leaf disease detection. The results demonstrate that while the Attention-based residual CNN model achieves a marginally higher accuracy of 98% compared to our proposed approach, it exhibits significant limitations in reasoning capabilities and lacks mobile/device deployment feasibility. In contrast, our proposed model attains 97.27% accuracy while providing enhanced functionality through disease classification and the generation of precise remedial recommendations, making it more suitable for practical agricultural applications.

**Table 4:** State-of-art comparison

Models	Reasoning	No. of	Dataset	Accuracy	Mobile/Device	Reference
	Capability	Classes			Deployment	
LeNet	No	10	PlantVillage	94–95%	No	[38]
AlexNet	No	10	PlantVillage	94–95%	No	[57]
Attention based residual CNN	No	98	PlantVillage	98%	No	[58]
AlexNet, SqueezeNet 1.1	No	10	PlantVillage + Self	96.3%	Yes	[59]
MobileNets	No	10	Self	90.3%	Yes	[60]
DCNN	No	6	Self	76%	No	[61]
ResNet50 baseline	No	7	PlantVillage	97.0%	No	[62]
Mask R-CNN	No	7	Self	78%	No	[63]
Inception + CBAM	No	8	Plantvillage	95.2%	No	[64]
BLIP-2 - LoRA (Ours)	Yes	10	PlantVillage + Self	97.27%	Yes	Our Proposed approach

### Experimentation of TLDVLM based portable device

**Fig. 9:**
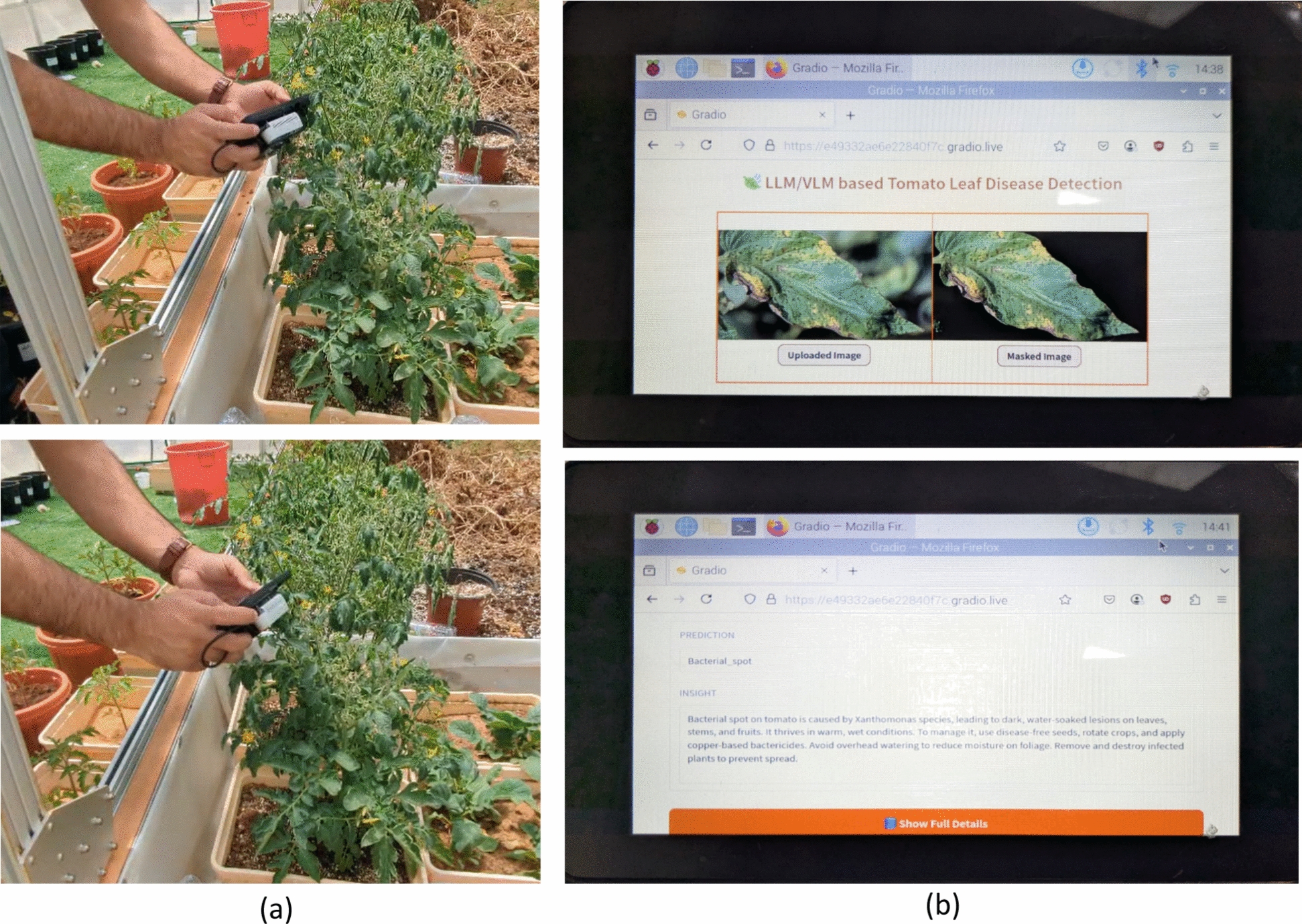
Proposed TLDVLM implementation: **a** Portable device testing at greenhouse, and **b** Device screen results

The validation of the TLDVLM model on portable device was carried out at greenhouse setting for tomato crop. The device is tested on tomato leaves to capture the images and then used as input for TLDVLM to analysis and generate the report. The final report is generated as PDF format which is further used for disease analysis and plant health monitoring. Figure  9 shows the experimentation at greenhouse test field.

## Conclusion

This study successfully developed and evaluated the Tomato Leaf Disease Visual Language Model (TLDVLM), a robust and efficient solution for the precise classification of 10 distinct tomato leaf diseases. By leveraging the advanced multimodal architecture of BLIP-2 and implementing Low-Rank Adaptation (LoRA) on its Q-Former, the TLDVLM achieved superior performance compared to traditional computer vision models like ConvNeXT-tiny and other parameter-efficient VLM adaptations such as CLIP-LoRA. The integration of GroundingDINO and SAM-2 for automated leaf segmentation proved crucial in enhancing the model’s focus and accuracy by eliminating irrelevant background noise.

Our experimental results unequivocally demonstrate the efficacy of the TLDVLM, with an impressive overall accuracy of 97.27%, complemented by high precision (0.9587), recall (0.9789), and F1-score (0.9681). This performance underscores the capacity of LoRA-adapted Vision-Language Models to retain powerful pre-trained knowledge while being efficiently fine-tuned for specific, high-stakes tasks like agricultural disease detection. The detailed analysis of class-specific performance revealed the model’s strong discriminatory abilities, with perfect classification on several disease types and effective handling of class imbalance through weighted sampling and loss functions.

Beyond its classification prowess, the TLDVLM’s practical utility is further enhanced by its deployment within a portable application. This tool provides instant visual feedback (raw and segmented images), disease predictions, and, critically, offers on-demand access to comprehensive information regarding disease causes and remedies via external knowledge bases (e.g., OpenAI). The ability to download a PDF summary for offline use signifies a significant step towards accessible and actionable agricultural diagnostics for farmers and growers.

In conclusion, the TLDVLM represents a promising advancement in intelligent agricultural systems, showcasing that state-of-the-art vision-language models, when combined with parameter-efficient fine-tuning techniques and smart integration, can yield highly accurate, robust, and deployable solutions for critical challenges like crop disease management. The TLDVLM model, while achieving high accuracy in tomato leaf disease classification, faces several key limitations that constrain its real-world applicability. The model’s training on primarily greenhouse-captured images and the PlantVillage dataset limits its generalizability to challenging outdoor field conditions characterized by variable lighting, occlusion, and background interference. Additionally, the current focus on static image analysis overlooks the potential for temporal disease progression monitoring. To address these limitations, future research directions include expanding the training dataset with diverse field-acquired images across multiple seasons and geographical locations, incorporating temporal modeling capabilities for early-stage disease prediction, and implementing domain adaptation techniques to enable model transfer across different crops and disease categories without requiring complete retraining.

## Data Availability

No datasets were generated or analysed during the current study.
